# Effect of Dropping Height on the Forces of Lower Extremity Joints and Muscles during Landing: A Musculoskeletal Modeling

**DOI:** 10.1155/2018/2632603

**Published:** 2018-07-02

**Authors:** Wenxin Niu, Lejun Wang, Chenghua Jiang, Ming Zhang

**Affiliations:** ^1^Yangzhi Rehabilitation Hospital, Shanghai Sunshine Rehabilitation Centre, Tongji University School of Medicine, Shanghai 201619, China; ^2^Department of Rehabilitation Sciences, Tongji University School of Medicine, Shanghai 201619, China; ^3^Sport and Health Research Center, Physical Education Department, Tongji University, Shanghai 200092, China; ^4^Department of Biomedical Engineering, The Hong Kong Polytechnic University, Hong Kong

## Abstract

The objective of this study was to investigate the effect of dropping height on the forces of joints and muscles in lower extremities during landing. A total of 10 adult subjects were required to landing from three different heights (32 cm, 52 cm, and 72 cm), and the ground reaction force and kinematics of lower extremities were measured. Then, the experimental data were input into the AnyBody Modeling System, in which software the musculoskeletal system of each subject was modeled. The reverse dynamic analysis was done to calculate the joint and muscle forces for each landing trial, and the effect of dropping-landing on the results was evaluated. The computational simulation showed that, with increasing of dropping height, the vertical forces of all the hip, knee, and ankle joints, and the forces of rectus femoris, gluteus maximus, gluteus medius, vastii, biceps femoris and adductor magnus were all significantly increased. The increased dropping height also resulted in earlier activation of the iliopsoas, rectus femoris, gluteus medius, gluteus minimus, and soleus, but latter activation of the tibialis anterior. The quantitative joint and muscle forces can be used as loading conditions in finite element analysis to calculate stress and strain and energy absorption processes in various tissues of the lower limbs.

## 1. Introduction

Landing is a common and important form of movement that is necessary in a variety of sports, dancing, and special occupations [1–5]. Landing is also very easy to cause injuries, especially on the lower limbs. About 49%–52% of the injuries in gymnastics training occur during the landing phase [1, 2]. The reasonable protection of landing injuries, as well as the clinical treatment and rehabilitation of patients after injury, requires a scientific understanding of the landing injuries.

Traditionally, some people thought that greater ground reaction force (GRF) or shorter time to peak vertical GRF (TPvGRF) would be more likely to incur damage [5, 6]. We found that, compared to women, men had significantly higher vGRF and rate of loading (ROL, i.e., vGRF/TPvGRF) [7–10]. In accordance with the above traditional notion, the phenomenon of women more prone to landing injuries could not be explained [11, 12]. Based on previous studies, we concluded that (1) the relationship between the ankle joint activity and the risk of injury was insignificant, while the joint angular velocity is positively related to the risk of injury [7, 8, 13]; (2) compared with women, men were more adept at using the ankle dorsiflexor and had better explosive strength and cocontraction of the ankle plantarflexor and dorsiflexor, and then, these factors induced the higher injury rate for women [8, 9]; and (3) the ankle brace significantly improved GRF and the muscle forces and enhanced the proprioception of the lower limbs [9, 10]. During landing, if the joint range of motion (ROM) was controlled within the tolerance range through muscle forces, more kinetic energy would be converted through increasing GRF to avoid excessive joint motions. This mainly reflects the relationship of muscle force, GRF, and joint kinematics. Therefore, the influence of muscle force must be considered in the research of landing biomechanics.

Electromyography (EMG) has been commonly used to reflect the muscle activity [7–9, 14, 15]. However, due to many influential factors, it is hard to accurately get the muscle force and torque based on EMG measurement. In recent years, the rapid development of computer technology brought about reverse dynamic analysis to a complex computational musculoskeletal model [16–18]. This provides a reasonable way for us to calculate muscle forces accurately.

Therefore, the purpose of this study was to evaluate effects of dropping height on muscle forces and force of the lower limb joints during landing using a computational musculoskeletal model and experimental data.

## 2. Materials and Methods

Based on our previous data [4, 7–9], a power analysis revealed that to achieve 80% statistical power, with an exploratory α level of 0.05, a minimum of 10 subjects were required. Thus, 10 subjects (6 women and 4 men) were recruited for this study. Their mean ± SD age was 23.8 ± 3.9 years, and the height and body mass were 165 ± 5 cm and 57.8 ± 8.5 kg, respectively. All subjects were right-leg dominant, which was determined individually by asking which one leg they would use to kick a ball as far as possible [7]. All subjects were physically active and had never experienced surgery, had no trauma and neurological dysfunction at least 6 months before the test, and were free from any trouble with inner ear problems, vision, neuromuscular dysfunction, or any orthopedic conditions. All subjects signed the University-approved informed consent before participating.

**Figure 1:**
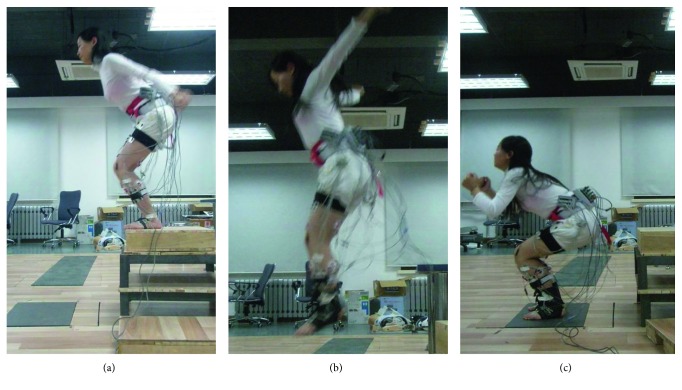
A female subject was in the trial: (a) jumping; (b) prelanding; (c) postlanding.

The subjects jumped from three different heights (32 cm, 52 cm, and 72 cm) and landed on a force plate (FP4060-08, Bertec Corp, Columbus, OH) with a half-squatting posture (Figure 1). These heights were determined according to our previous studies [4, 7–9, 13] and a study by Mcnitt-Gray [19]. The landing posture was also defined elsewhere as a simulated parachute landing in China [8, 9]. The subjects were instructed to takeoff and touch down with both feet, to lean forward with body at takeoff, to make a half-squatting posture with foot contact, and finally to break the fall smoothly. The trial order was random to avoid the order effect on the results.

The GRF data in three directions were collected at a sampling frequency of 1000 Hz. An Optotrak Certus motion capture system (Northern Digital Inc., Waterloo, Canada) was used to measure the limb kinematics during landing. Each body segment was registered using a plate with 4 noncollinear LED markers, which was tightly attached to the corresponding segment [8, 9]. These markers were captured, and the kinematic data were then analyzed using Visual3D (C-Motion Inc., Rockville, MD). The processed signals were synchronized by an analog-to-digital converter.

The anthropometric data (body weight, body height, pelvis width, thigh, shanks, and foot length) were measured from each subject and then were used to construct the musculoskeletal model in the AnyBody Modeling System version 5.0 (AnyBody Technology A/S, Aalborg, Denmark) [20]. The model was developed from the Twente Lower Extremity Model (TLEM) in AnyBody Managed Model Repository [21]. This model is based on a comprehensive and consistent data set from one donor [22]. According to our anthropometric measurement, the model was then scaled with a mass-fat scaling algorithm to simulate each individual. The AnyBody Modeling System and the musculoskeletal model have been validated to be useful for analyzing human movement [20]. The main input of the modeling is the kinematic and GRF data from the subjects while landing. The GRF was filtered using a Woltring filter that does not affect the timing and hardly affects the amplitude.

The inverse dynamic analysis calculated the force of each muscle bundle and the lower limb joints. Because the force directions were similar for the bundles in the same muscle, we combined them all for simplifying the analysis. The calculated forces of the hip, knee, and ankle joints were normalized to the body weight (BW) of each subject. The effect of dropping height on these force values was evaluated using the univariate analysis of variance (ANOVA) and Turkey's HSD post hoc analysis. The statistical analysis confidence interval was 95%, and the statistical analysis was performed using the free data analysis system VassarStats (http://vassarstats.net/) [8, 9].

## 3. Results and Discussion

### 3.1. GRF and Joint Force

**Figure 2:**
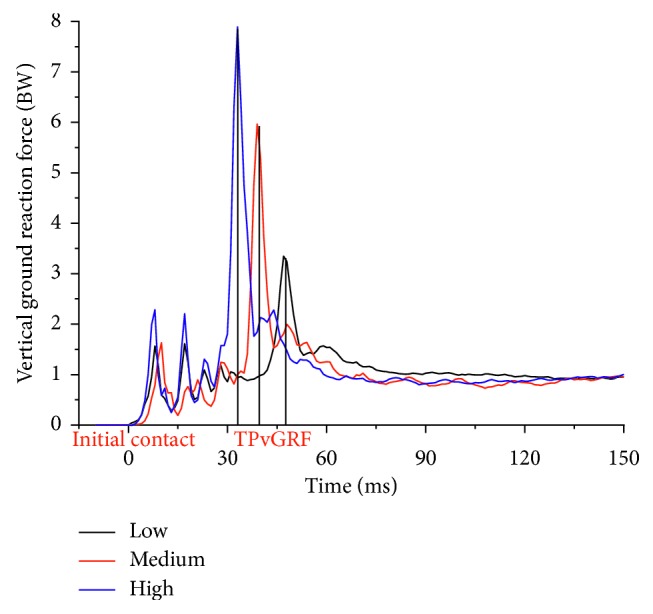
Vertical ground reaction forces when a representative subject landed from three different heights.

As a critical factor during landing, the dropping height greatly affects the landing speed. When landing from three different heights of 32 cm, 52 cm, and 72 cm, the performer at initial contact had the average speed of 2.1 m/s, 2.3 m/s, and 3.0 m/s, respectively, with significant statistical differences (*P* < 0.001). As shown in Figure 2, with increase of the dropping height, peak vGRF increased significantly (*P* < 0.001).

We have found a linear relationship between the peak vGRF and root dropping height [10]. In the present study, we found that, with the increase of the dropping height, the GRF peak increased significantly in the vertical and anterior-posterior (A-P) directions, but the dropping height has no significant influence on the peak medial-lateral (M-L) GRF peak, TPvGRF, or ROL. This is consistent with the finding by Yeow et al. [23].

Effects of dropping height on GRF were further reflected in the similar effects on the contact force of lower limb joints. This would lead to injury risk of these joints during high-speed landing. As listed in Table 1, with increasing of the dropping height, the vertical force of each joint significantly increased. The influence of dropping height on the ankle joint in any horizontal direction was not significant. Though no significant influence was found in the dropping height on the knee joint force in the A-P direction, the force in the M-L direction was significantly higher while landing from higher positions. We also found significant influences of the dropping height on the force of the hip joint in all three directions.

This study showed that the force peaks of the ankle joint and the hip joint could reach more than 20 BW when subjects landed from 72 cm height. If the dropping height was even increased, higher joint force may lead to injuries. Therefore, the high peak joint force was reasonable in the current study. When subjects landed from low and medium heights, from the ankle to the knee and the hip joints, the peak forces in the vertical direction declined. However, when subjects landed from 72 cm height, the vertical force peak of the hip joint was significantly higher than that of the knee joint. It may be caused by higher muscle force of gluteus during landing from higher level.

### 3.2. Muscle Force

Because the joint force is directly related to the joint torque and the muscle force, the effect of the dropping height on the joint force is further reflected in the force of each muscle. In the current study, the same subject had similar musculation pattern even while landing from different heights, but certain difference of the sequence of muscle activation existed among different subjects.

**Figure 3:**
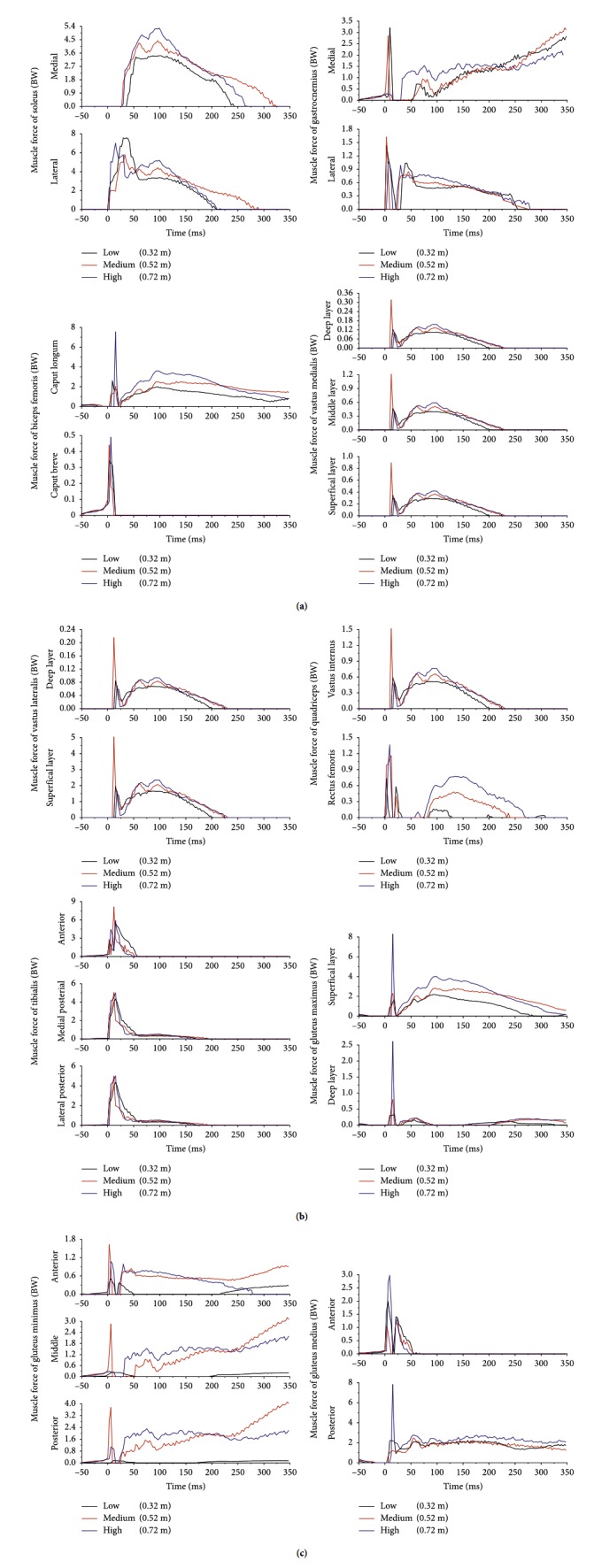
Forces of leg muscles when a representative subject landed from three different heights.

Figure 3 showed the muscle activation of a representative subject landing from three heights. The peak forces of main muscles were statistically analyzed and shown in Table 2. With increasing of dropping height, the peak forces increased for most muscles in the lower limb, in which the rectus femoris (RF), GMax, gluteus medius (GMed), vastii, biceps femoris (BF), and adductor magnus all showed significant changes.

The increased dropping height also resulted in earlier activation of the iliopsoas, RF, GMed, gluteus minimus (GMin), and SOL, but latter activation of the tibialis anterior (TA). In addition, when subjects landed from higher positions, the time from initial contact to peak force of RF and SOL was significantly longer. The longer the duration of muscle activity, the longer and more durable the muscle force used to counter the impact.

As seen in Figure 1, we divided the entire landing process into three phases. Because the researcher instructed subjects not to jump higher than their initial level, the timing and amplitude of each muscle had consistent activity pattern and amplitude. The flying phase can also be called as the prelanding phase, while the last phase can be called the postlanding phase. Some studies showed that, with increasing of dropping height, the EMG onset latency and duration would be longer in TA, soleus (SOL), RF, and BF, but the prelanding EMG duration was less affected by the change of the dropping height [24, 25]. Our previous study also showed the similar phenomenon in TA and gastrocnemius (Gast) [7–9]. Because the landing is a high-intensity impact action, the requirement for muscle energy is high. Even during a lower height landing, the muscle will be prepared with enough time. If the dropping height was increased, the accumulation of muscle power is not through the duration, but through the activity amplitude.

The calculated results showed that, with increasing dropping height, the maximum force of TA increased, but there was no significant difference between different heights. This was consistent with the experimental results. This also showed that ankle plantarflexor has a more important role on the landing movement than the dorsiflexor. In addition, the force of triceps muscle was up to 10 BW level and had a main role in changing the mechanics of the shank.

As for the effects of dropping height on the knee flexors and extensors, various experimental studies gave different findings [26–29]. This study showed that the knee flexors and extensors were all significantly activated to maintain the balance of the knee joint. As a result, when the dropping height was higher, the compressive force of the knee joint also increased because the larger muscle forces provided additional loads.

During landing, the muscle contraction mode is very complex, and the forces of some muscles or muscle groups are even beyond the level of ground reaction force. Because these muscle forces would be loaded on the skeleton around joints, they may have great impact on the stress or strain of bone and cartilage, energy absorption, and transmission in the lower limbs. Some authors have tried to get these data using finite element analysis [30], but it is necessary to understand the muscle contraction mode and force before modeling of local joints or organs. This study could provide more precise loading conditions for future finite element simulation of any phase of a typical landing movement.

## 4. Conclusion

Based on the experimental data, the inverse dynamic model of the human musculoskeletal system was established in this study. The force of the lower limb joints and the muscle groups was calculated while the subjects landed to evaluate the effects of dropping height on these parameters. The quantitative joint and muscle forces can be used as loading conditions in finite element analysis to calculate stress and strain and energy absorption processes in various tissues of the lower limbs. This would be useful for further understanding of the injury mechanism during landing.

## Figures and Tables

**Table 1:** Effects of dropping height on the forces of the ankle, knee, and hip joints (unit: body weight, BW).

Joint	Direction	Low (32 cm)	Medium (52 cm)	High (72 cm)	*F*	*P*
Ankle	Vertical	16.95 ± 2.59	18.88 ± 3.46	23.98 ± 4.21	8.815	<0.001
	A-P	4.87 ± 0.96	5.19 ± 1.04	5.18 ± 1.11	1.562	0.252
	M-L	4.17 ± 0.88	4.23 ± 1.06	5.83 ± 0.96	2.204	0.147

Knee	Vertical	9.09 ± 2.09	11.5 ± 2.26	14.58 ± 2.17	6.895	0.004
	A-P	2.86 ± 0.74	5.23 ± 1.09	4.98 ± 1.32	1.785	0.204
	M-L	0.72 ± 0.31	0.75 ± 0.24	0.88 ± 0.32	7.307	0.007

Hip	Vertical	8.50 ± 2.53	9.49 ± 2.68	22.81 ± 4.69	10.208	<0.001
	A-P	3.49 ± 1.15	4.08 ± 1.68	11.9 ± 2.96	8.024	0.003
	M-L	3.74 ± 0.69	4.76 ± 1.12	12.56 ± 3.36	8.749	0.004

A-P: anterior-posterior; M-L: medial-lateral.

**Table 2:** Effects of dropping height on the peak forces of main muscles in the lower limbs (unit: body weight, BW).

Muscles	Low (32 cm)	Medium (52 cm)	High (72 cm)	*F*	*P*
Gastrocnemius	5.3 ± 1.6	5.8 ± 1.7	5.5 ± 2.0	0.21	0.812
Soleus	9.9 ± 3.0	10.6 ± 3.7	11.8 ± 3.8	0.75	0.482
Tibialis posterior	4.1 ± 1.7	4.8 ± 2.5	3.5 ± 1.6	1.09	0.351
Tibialis anterior	7.2 ± 1.5	7.6 ± 1.7	7.6 ± 1.8	0.19	0.828
Biceps femoris	3.3 ± 1.2	4.0 ± 1.1	5.6 ± 1.9	6.75	0.004
Rectus femoris	0.5 ± 0.2	0.7 ± 0.3	1.0 ± 0.4	5.14	0.013
Vastus medialis	0.7 ± 0.2	0.9 ± 0.3	1.0 ± 0.3	4.99	0.014
Vastus lateralis	1.7 ± 0.5	2.7 ± 0.9	3.2 ± 1.1	7.84	0.002
Vastus internus	0.4 ± 0.1	0.7 ± 0.2	0.8 ± 0.3	6.36	0.005
Gluteus maximus	2.5 ± 0.8	5.1 ± 2.6	9.7 ± 2.5	28.78	<0.0001
Gluteus medius	3.8 ± 1.3	3.6 ± 1.6	8.5 ± 2.5	21.97	<0.0001
Gluteus minimus	0.7 ± 0.3	1.9 ± 0.9	2.8 ± 0.8	21.59	<0.0001
Iliacus	1.3 ± 0.4	1.4 ± 0.6	1.3 ± 0.5	0.40	0.67
Adductor magnus	1.6 ± 0.5	1.8 ± 0.6	2.8 ± 0.7	11.00	<0.001
